# Putting an Integrated Theoretical Framework of Student Engagement into Practice: A Case Study of Three Student Initiatives at the Technical University of Munich

**DOI:** 10.1007/s40670-024-02223-5

**Published:** 2024-11-18

**Authors:** Johannes Reifenrath, Anna Buchner, Antonia Leeb, Lina Hermann, Christine Allwang, Andreas Dinkel, Pascal O. Berberat, Marjo Wijnen-Meijer

**Affiliations:** 1 School of Medicine and Health, Technical University of Munich, TUM Medical Education Center, Ismaninger Str. 22, Munich, 81675 Germany; 2 School of Medicine and Health, Technical University of Munich, Student Council of Medicine, Ismaninger Str. 22, Munich, 81675 Germany; 3 Department of Psychosomatic Medicine and Psychotherapy, Klinikum rechts der Isar, TUM University Hospital, School of Medicine and Health, Technical University of Munich, Ismaninger Str. 22, Munich, 81675 Germany; 4 Institute of Medical Education, Medical Faculty and University Hospital Carl Gustav Carus, TU Dresden University of Technology, Fetscherstraße 74, Dresden, 01307 Germany

**Keywords:** Student engagement, Medical education, Curriculum, Medical schools, Faculty

## Abstract

Student engagement is perceived as an increasingly important but complex phenomenon in medical education. Recently, integrated theoretical frameworks have been introduced to conceptualize student engagement from a psychological, behavioral, and psychosocial perspective. The body of literature underpinning the development of the frameworks is enormous, but there is a relative paucity of reports detailing the use of the frameworks in practice. Here, we present a case study of three de novo student initiatives at the Technical University of Munich. The initiatives cover various topics, from case-based learning tutorials to extracurricular offerings on mental health and mentoring. We will map each project to the integrated framework proposed by Kassab et al. (*Med Teach* 45:949–965, 2023) and assess its usefulness in capturing student engagement. We conclude that the framework holistically describes the engagement profile of each project but does not consider the different roles students assume when participating in a project or incentivizing it.

## Introduction

Student engagement (SE) has become an increasingly relevant and researched topic. Its importance and benefits for students’ academic success and well-being have become evident [1]. There are positive impacts on students’ learning process, the school’s governance, and the curriculum [2, 3]. Though the concept of SE might seem intuitive at first, there have been several distinct approaches to defining student engagement [4–9].

The first concepts of SE focussed on students’ behavioral patterns within the educational system and emerged from the field of pedagogy in secondary education. Faced with absenteeism and low participation levels, Natriello (1984) defined SE as the “participation in activities offered by the school” [4]. The behavioral perspective is also widely accepted in higher education due to its intuitiveness and measurability [10]. SE also includes the representation of students in institutional committees [5], their active participation in educational activities, and the time and effort invested in it [6]. From these definitions, it becomes clear that the behavioral perspective subsumes student and institutional efforts but does not sufficiently engage the student’s own psychological state [10].

The behavioral perspective is, therefore, complemented by the psychological one. While the psychological concepts differ in the items analyzed and their complexity, they generally suggest the existence of distinct dimensions within the perspective [10]. Educators distinguish between the behavioral dimension (conduct, involvement in learning, participation in educational activities), the cognitive dimension (self-regulation and use of deep learning strategies), and the emotional dimension (affect) [8]. In 1989, Finn introduced the participation-identification cycle as one of the first psychological concepts considering the emotional component, where participation, academic achievement, and identification with the educational institution boost each other [7]. It is apparent that, from the psychological perspective, dimensions are interconnected, leading to the emerging concept of antecedents of SE, i.e., factors that promote or mediate SE.

Over the past decade, so-called integrated frameworks have been proposed that merge the behavioral, psychological, and sociocultural perspectives into a holistic approach [8, 9, 11]. One of the most recent and comprehensive frameworks was presented by Kassab et al. (2022) [8]. Elaborating on earlier integrated frameworks, it defines five components for SE specifically applied to health professions education. The first component covers the antecedents of engagement, referring to institutional and student factors and their interactions. Kassab’s framework incorporates earlier findings on psychological needs such as competence, such as a sense of confidence and effectiveness, a sense of belonging, and the social integration of a student. They are shown to significantly positively affect student engagement [12]. The levels at which students experience belonging can affect engagement, quality of social interactions, and academic performance [13]. SE is further influenced by a student’s own learning disposition and personal factors, including motivation, emotion, self-regulation, and self-efficacy [1, 14]. The second component in Kassab’s framework lists mediators of engagement, namely self-efficacy, motivation, belonging, and reflectivity. In the third component, five dimensions of engagement are identified: cognitive, behavioral, emotional, agentic, and sociocultural dimensions. However, the particular value of Kassab’s framework lies in its fourth component. In addition to earlier works, it introduces two main spheres of engagement: engagement in one’s own learning and engagement through partnerships with the university. The latter can be subdivided into the provision of the education program, scholarly research, governance and quality assurance, and community activities. Lastly, the fifth dimension refers to the outcomes of engagement. [8]

Kassab’s third component, the five dimensions of engagement, is closely related to the multi-dimensional theory of SE [14, 15]. It posits that student engagement happens on a behavioral, emotional/affective, and cognitive dimension. Cognitive engagement represents students’ mental resources and willingness to understand complex issues and attain skills. This includes using deep learning strategies, deep concentration, thinking about learning activities, perceived value of academic tasks, and high-order skills. Behavioral engagement highlights participation in school activities and observable academic performance, involving attendance, effort, persistence, class, extracurricular activities, and attentiveness to educational activities. Emotional engagement emphasizes the positive and negative emotions and feelings of bonding students experience about aspects of their academic life, including their peers, teachers, classroom, and school. These dimensions are interrelated and can be applied to multiple facets of the student experience [14, 16].

The theoretical frameworks can guide the assessment methods of student engagement. Self-report surveys are the most frequently used method to this end. Their major advantages include low-level administration and low cost. Depending on their design, they can be tailored towards measuring SE from the behavioral perspective, such as the National Survey of Student Engagement [10], or from the psychological perspective, such as the one-dimensional Learners’ Engagement and Motivation Questionnaire [17]. Most available questionnaires assess SE through more than one perspective. Despite their use, questionnaires are limited in their ability to represent the complex and dynamic nature of SE. More objective methods, such as observation of student behavior or real-time measures, have been established but are generally not used on large cohorts.

The general endorsement of SE by medical educators has sparked a new discussion on how to promote it. Some instructional methods can enhance medical student engagement by targeting one or more of the following driving factors: positive student-peer and student-faculty relationships, enhancing students’ sense of competence, agency, empowerment, and perceived relevance through meaningful learning activities [14]. Another key driver for SE is a general positive regard for students that fosters psychological safety. Specific instructions on this can be cross-referenced in the AMEE guide Nr. 152 [8]. Briefly, the first step towards a supportive culture is a general appreciation of different personal and sociocultural backgrounds. Further steps include integrating new students through student groups and institutional-level groups, as well as providing informational support through specific orientation, peer support programs, and financial aid, particularly for disadvantaged students. Psychological safety can be achieved by rewarding students who speak up and making teachers approachable to discussions.

Finally, SE is enhanced through a collaborative organizational culture that provides visible opportunities for engagement. Freitas et al. (2023) found that the capacity of students to participate in extracurricular activities and the school’s values also impact engagement [18]. The findings suggest that institutions need to make engagement strategies visible and accessible to students and to involve both students and staff in championing engagement [18].

A condensed version of specific methods has been presented by Peters et al. (2018) [19]. In their twelve tips for enhancing SE, the authors cover different aspects, such as the culture and framework for student engagement, the importance of feedback, student involvement in educational activities, and the implementation of specific structures for student engagement. This can be achieved by creating defined roles, offering opportunities, empowering students with real responsibility, and genuinely valuing the students and their opinions to allow them to leave a meaningful impact at their schools and prevent student disengagement and isolation [3, 20].

Most integrated theoretical frameworks were conceived through an inferential approach, where study and literature findings were synthesized into the framework. However, there is a relative scarcity of reports that reflect real-life student initiatives through the frameworks. To supplement this lack of practical application, we explore three use cases of SE at the Technical University of Munich (TUM) through the theoretical framework proposed by Kassab et al. [8]. The described student initiatives comprise a tutoring initiative to supplement curricular teaching and two extracurricular initiatives on mental health and mentoring. We will describe each component in the context of the projects and discuss the usefulness of the theoretical framework in capturing student-led initiatives.

## Examples of Projects

### Integrated Clinical Case Discussions — a Peer Teaching Initiative in Internal Medicine

One example of successful student contribution to the curriculum is the implementation of the Integrated Clinical Case Discussions (ICCD) at the TUM [21]. They are a peer-organized series of case-based tutorials that track an interdisciplinary lecture on internal medicine, pathology, and surgery. There are twelve sessions per semester in the first and second years of students’ clinical training.

**Fig. 1:**
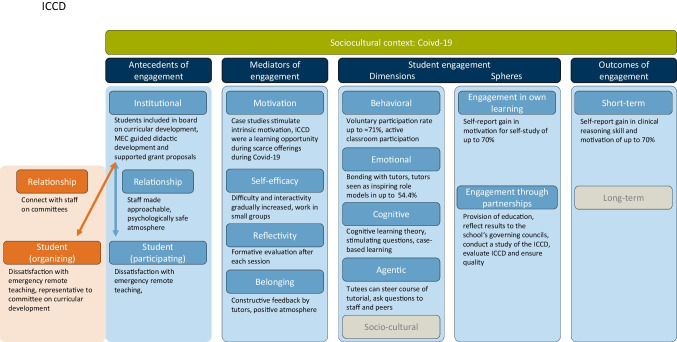
Case study of ICCD through Kassab’s theoretical framework of SE [8]. Green: sociocultural context, dark blue: components of SE, light blue: explanation of each component in the context of ICCD from the perspective of a participating student (tutee), orange: antecedents of SE for organizing students, gray: items not further discussed in this paper

The project demonstrates the importance of a faculty’s collaborative organizational culture as an antecedent of meaningful SE (Fig. 1). The impetus for the project was provided by three undergraduate student representatives who, in response to poor ratings of emergency remote teaching, sought a way to offer additional learning opportunities while also alleviating pressure on faculty staff. In the school’s council on curricular development, the student representatives connected with staff from the TUM Medical Education Center, who guided the development of the didactic concept and helped secure funding for the project. The school continued to cover the administrative aspects of contracting tutors while the student representatives decided on financial expenses.

We have described the antecedents of engagement from the perspective of the incentivizing student representatives, but to understand the extent of SE, we must also analyze the ICCD through the perspective of participating tutees. Since the ICCD were implemented against the background of the emergency remote teaching during the COVID-19-crisis [22], many students missed meaningful interactions with instructors and patients and reported a loss in educational quality [23]. ICCD were therefore aimed at (a) providing additional learning opportunities by teaching clinical reasoning skills through interactive case studies and (b) improving the student-school relationships.

Case studies are a useful format for positively mediating engagement in higher education [24]. Since most emergency remote lectures were restricted to teaching facts and declarative knowledge, ICCD stimulated students’ intrinsic motivation by discussing patient cases. At the same time, ICCD considered tutees’ self-efficacy and feeling of competence by gradually increasing the level of interaction and difficulty (e.g., from taking the patient history to integrating the findings into a final diagnosis). At the end of each session, there was also time for reflection through an evaluation. In the definition of Kassab et al., belonging comprises of a feeling of being “valued, accepted, included, and encouraged by staff and peers “ [8]. In our study, this was reflected by a composite score for the category “educational atmosphere” of 1.35 on a 5-point Likert scale from 1 to 5 (*n* = 107) [21]. The exact study design can be cross-referenced.

ICCD also sought to improve the student-school relationships. In some ICCD sessions, both lecturers and students could meet and discuss cases together, moderated by a student tutor. In the end-of-class evaluations, we saw that this made teachers more approachable and afforded psychological safety for students.

We observed positive effects of ICCDs on participants’ engagement in all dimensions. The behavioral dimension can be quantified through participation rates in educational activities [8, 25]. With 44.5% of all eligible students having attended at least one session in the first semester and 71.0% in the second semester, we infer that ICCD gained significant traction in the student body. We also required tutors to employ cognitive learning theory and constructivist teaching, by asking stimulating questions to activate prior knowledge to engage participants cognitively. A self-report questionnaire was developed based on literature findings to capture students` learning experience from the psychological perspective. Participants also rated the tutors as excellent (average score of 1.26 on a 5-point Likert scale from 1 to 5; *n* = 121). In a qualitative analysis of student commentaries (*n* = 123), we also saw that students felt grateful for the tutorial and praised them. In 54.4% of evaluations, tutees felt emotionally connected to their tutors and identified them as inspiring role models. The role of different sociocultural backgrounds was acknowledged in the project but was not explicitly addressed [21].

As for learning outcomes, we observed significantly better subjective ratings of cognitive skills training and knowledge retention in the ICCDs in comparison to the lecture (*p* < 0.01). In fact, in a comparative self-assessment (CSA) [26], tutees reported up to 70.31% (*n* = 114) increase in the ability to interpret diagnostic findings after the tutorial. After the tutorial, students rated ICCDs as more motivational than the corresponding lecture and also said they felt more comfortable with peer instruction after the tutorial (CSA gain = 69.57%, *n* = 111). The study design did not allow for a long-term follow-up of engagement outcomes [21].

ICCD further showcase SE through partnership with the university. Not only did tutors deliver educational opportunities, but the student representatives also carried out a study of the tutorials, guided by the school’s staff. The results were presented to the council on curricular development and to the faculty board. In fact, ICCD and the two projects described below catalyzed the inception of a special student engagement fund by the faculty to financially support student initiatives.

### MIND your Health!

Studying medicine can be very demanding for students. Excessive workload, work-life balance conflicts, and system-level factors such as administrative failures contribute to the stress experienced by medical students [27, 28]. It has been shown that symptoms of burnout, depression, and anxiety, but also somatic complaints and alcohol misuse, are elevated in medical students compared to the general population [28–32]. Struggling with such problems can lead to a decrease in academic performance [33, 34]. However, many medical students are hesitant to seek professional help for mental health problems, mainly due to fear of stigmatization [29].

**Fig. 2:**
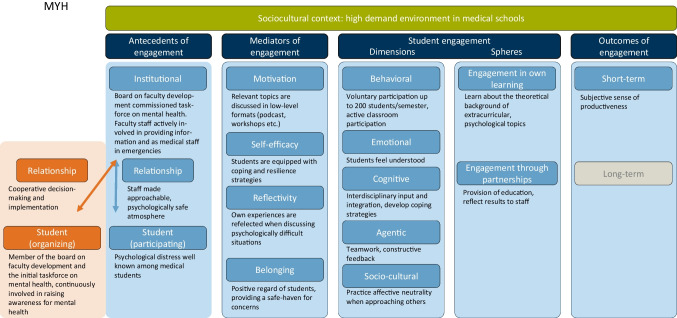
Case study of *MIND your Health!* through Kassab’s theoretical framework of SE [8]. Green: sociocultural context, dark blue: components of SE, light blue: explanation of each component in the context of MIND your Health! from the perspective of a participating student, orange: antecedents of SE for organizing students, gray: items not further discussed in this paper

To mitigate this situation, *MIND Your Health!* was founded in 2019. The starting point for this initiative was concerns voiced by representatives of the School of Medicine student council, who called for actions to destigmatize mental health problems (Fig. 2). Initially, the board of faculty development commissioned an interdisciplinary task force on mental health, which soon evolved into the project *MIND Your Health!* It comprised members of the student council, faculty members, and specialists from the Department of Psychosomatic Medicine and Psychotherapy at the Klinikum rechts der Isar and the TUM Medical Education Center. Working in conjunction with these institutions has played an instrumental role in providing critical resources, both structurally and psychosocially.

During their first meeting, the members decided on the aims of *MIND Your Health!* These are fourfold: (1) to raise awareness about mental health problems; (2) to provide information about mental health; (3) to incorporate the topic of mental health in teaching courses; and (4) to offer basic psychological support for medical students in need. While the academic and clinical staff are responsible for the last two goals, members of the student council have been very enthusiastic about the development of innovative formats to pursue the first two goals. These actions were supported and supervised by the faculty members of *MIND Your Health!*

To enable low-threshold access to the topic of mental health, *MIND your Health!* offers events, workshops, and a contact point for students. Each term, the working group organizes an event that reaches about 200 medical students. Examples include a poetry slam, a TedTalk, and a panel discussion. For instance, physicians talked about emotional closeness and distance in the medical profession or discussed how to deal with physical and mental illness while being a doctor. Also, a podcast called *Speak your MIND* was started in 2022. Here, speakers examine topics such as suffering from eating disorders or struggling with finding a fulfilling career. *MIND your Health!* has also aimed to improve physical and mental well-being. As low to moderate-intensity workouts seem to reduce stress and depressive symptoms [35], a running group and a yoga class were founded. Moreover, a peer-led facultative course called “Psychological First Aid” is offered for medical students who are interested in learning, in a simulated environment, how to help others who are experiencing acute stress after a critical event.

To successfully implement these projects, especially motivation and belonging, play an important role by mediating between institutional and student-level antecedents and the actual engagement. Projects are generally intended to extend a sense of appreciation and understanding towards students (belonging) while also addressing topics that students can relate to (motivation).

What is more, student engagement in this initiative manifests across various dimensions. Regarding Kassab et al. (2022), student engagement includes different dimensions [8]. For instance, students engaged in the “Psychological First Aid” workshop actively study the theory beforehand, take on leading roles, and navigate between fellow students, acting patients, psychologists, and the faculty. The organizing students create a hands-on project that combines the transfer of theoretical and practical knowledge on how to help reduce acute distress after a traumatic situation (behavioral); through their participation, fellow students confront sensitive topics around mental health (emotional); the integration of knowledge from medical school, personal experiences, and input from other professions requires an interdisciplinary approach to developing and applying psychological coping strategies (cognitive); students take on leadership roles, provide constructive feedback to peers, and practice teamwork (agentic); mental health awareness, being a broad and complex topic, concerns people from all backgrounds which requires openness and freedom of judgment (sociocultural). A vital aspect of the project is the students’ proactive involvement in their own learning. Students must engage in self-study to familiarize themselves with the relevant mental health topics. This preparation enhances their ability to effectively educate others, and their engagement deepens as they share knowledge and collaborate with fellow students. Partnerships with the Medical Education Center, the Department of Psychosomatic Medicine and Psychotherapy, and the student council ensured professional oversight and financial and logistic support.

### MED ME — Mentoring for Medical Students

MED ME is a peer-organized mentoring program that connects students with peers, doctors, and researchers.

Four to six first-year clinical students are matched with a senior peer and a physician-mentor based on specialty interest. At TUM, all first-year clinical students are new to the campus and have completed their preclinical training at a different university.

During the semester, three meetings take place. The first one is scheduled right after the semester starts, allowing the group members to get to know each other and discuss potential lab visits or clinical internships. Additionally, the administrative questions on the clinical studies, e.g. the exams, are addressed by the peer. The second meeting is scheduled in the middle of the semester and can be held in a more personal atmosphere. Topics are learning strategies and the group’s individual focus, e.g., on the dissertation, internships, training practical skills, and studying abroad. The third meeting at the end of the semester is used to evaluate the process and to discuss the semester. After three sessions, the groups can be continued if the students, peers, and mentor wish to do so. However, the official structure of MED ME ends at this point.

**Fig. 3:**
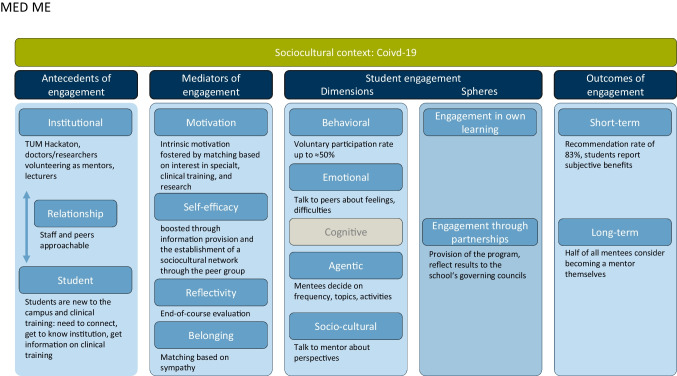
Case study of MED ME through Kassab’s theoretical framework of SE [8]. Green: sociocultural context, dark blue: components of SE, light blue: explanation of each component in the context of MED ME from the perspective of a participating student (mentee), gray: items not further discussed in this paper

The matching process of MED ME is crucial to the success of the project and is genuinely guided by the psychological mediators of SE (Fig. 3). It is aimed at fostering participants’ intrinsic motivation and sense of belonging by considering different interests in specializations, interest in research versus clinical work, and sympathy towards mentors and peers. In the semester’s final meeting, the project provides time for a thorough reflection and evaluation.

In well-functioning mentoring groups, multiple dimensions of SE are addressed throughout the program (Fig. 3). MED ME empowers mentees to actively shape their experience by deciding on the frequency of the meetings and suggesting discussion points and activities (feeling of agency). Mentees can exchange views and expand their perspectives, especially in the scheduled conversations with the mentors. At the same time, MED ME provides a supportive platform where mentees can share feelings and obstacles they face in their immediate studies, fostering a sense of understanding and support. From a behavioral perspective, we noticed great interest in the project, with approximately 50% of newly enrolled students participating as a mentee (145 out of approx. 300). It is important to consider that MED ME was implemented amidst the COVID-19 pandemic. In the subsequent semesters, we noted a slightly declining interest; however, the number of participants remained above 100 in the following semester.

Regarding the institutional antecedents for facilitating SE, our school helped develop the mentoring program through the TUM Hackathon, a program during which students can research and develop different ideas from computer science to medical students. The mentoring project used this program to create the website for the booking process. Every semester, new mentors from different specializations are recruited. This is key to a successful continuance of the project. To do so, the mentoring project is presented and advertised in various committees of the clinic and medical faculty, as well as at welcoming events for new students at the university. Staff also supports MED ME by organizing a lecture on non-classic career paths in medicine with, for example, doctors without borders, doctors in mountain rescue, or prison doctors.

Summative evaluations were administered to measure the outcomes of the first three mentoring cohorts. They show that more than 83% of all mentees (*n* = 71) recommend the program to other students. We believe this underscores and corroborates our conceptual approach to mentoring and the matching of groups. Most students say they have profited a lot or well from the program. Only around 6.4% claim to have had no profit, while around half of the mentees can imagine themselves becoming a peer the following semester. This suggests a potential long-term outcome on personal development: that students participate first as mentees, later as peers, and eventually as doctors or researchers.

## Conclusion

In this monograph, we first outlined the theoretical underpinnings of SE in medical education. We then put the concepts of SE into practice by mapping three de novo student initiatives at TUM onto one of the proposed frameworks. For this, we chose the integrated framework developed by Kassab et al. [8] because of its emphasis on SE through partnership with the university, which is a prominent feature exhibited in all described projects.

Our case study suggests that successful student initiatives are built on the joint cooperation of faculty, staff, and student leaders. At TUM, there was a collaborative culture [18], as shown by the supportive roles faculty staff played in planning, implementing, and evaluating each project. On the other hand, there were students committed to implementing their projects. These organizing students were mostly members of the school’s planning committees, highlighting the need to include students in the formal bodies of school governance to allow student initiatives [5, 19].

In our study of three student initiatives, we have described the antecedents of engagement not only for the incentivizing students but also for the participating students. They range from dissatisfaction with learning opportunities to distress and a need for belonging, corroborating previous literature on student experiences [23]. Ultimately, it becomes apparent that the antecedents of participants are clearly distinct from the antecedents of the organizing students. To accommodate these differences in antecedents, we suggest the introduction of the student’s position to the framework: participating students or the organizing student. While the framework was developed to assess SE in general [8], we believe the addition of distinct positions is an important feature for the specific assessment of student initiatives. Interestingly, these positions also reflect the 12 + 1 roles of medical students previously identified by Karakitsiou et al. [36]. Out of them, six roles refer to students as information receivers, educational resource consumers, and classroom facilitators. This corresponds to the position of students as participants in the theoretical framework of SE. The other half of roles describe students as educational planners and assessors as well as role models. In the theoretical framework, this is reflected in the position of students as organizers.

All projects rely on all four mediators, albeit, understandably, to different extents. Conceptually, self-efficacy was the most prominent in the ICCD, while the contextual mediators (reflectivity and belonging) were more visible in the other projects. Depending on the type of curricular or extracurricular activity, we have seen that the dimensions of engagement show a project-specific profile. Among the three initiatives, the ICCDs are the only ones directly supporting a curricular lecture and are primarily invested in the cognitive and behavioral dimensions of engagement. The other two initiatives offer extracurricular activities that place a special focus on the emotional and sociocultural dimensions of engagement. We conclude that for each project, a signature profile can be established within Kassab’s framework, where certain aspects are pronounced. This again reflects the framework’s value as it can be extended to diverse SE projects with no loss in applicability [8].

Finally, the unique value of the framework lies in the sphere of engagement through partnership with the university. As described, the projects are involved in the provision of education, research, and quality assurance. Importantly, due to the positive reception of these projects and the trust built between faculty and students, the school’s dean dedicated an annual budget to be administered by the student council to foster student initiatives in education. We believe this to be a flagship example of empowering students to impact their school’s governance and to support student engagement.

As the academic environment changes, questions such as why and how to engage students become increasingly important [14]. Student engagement is a multi-dimensional concept that is often hard to grasp for teachers and students, but its relevance is evident. As medical students can profit from student engagement in medical schools in many ways, it is vital to implement and enhance opportunities for student engagement to enrich teaching and shape positive learning environments during medical education [19]. Influencing factors such as teachers, student motivation, and other intrinsic factors, as well as academic belonging and resilience, should be considered in attempts to create more engaging content and class activities. More research is still needed on the topic, but there are already promising approaches to understanding and improving engagement, which can help educators to enhance teaching effectiveness and students’ learning experiences.

## Data Availability

The data that support the findings of this study are available from the corresponding author upon reasonable request.

## Abbreviations

SE: Student engagement
AMEE: Association for Medical Education in Europe
TUM: Technical University of Munich
ICCD: Integrated Clinical Case Discussion
